# Supplementary material for: TRAF3 suppression encourages B cell recruitment and prolongs survival of microbiome-intact mice with ovarian cancer
Source: J Exp Clin Cancer Res. 2023 May 1;42:107. doi: 10.1186/s13046-023-02680-7 (PMC10150478; doi:10.1186/s13046-023-02680-7)
Supplement: Supplementary file 1 — Additional file 1: Fig. S1. Establishing a library-expressing cells. A WT mouse harboring ITB1-related ascites and small tumors, indicated by the white arrows or the black arrows in the IHC-stained tissues. MLN- mesenchymal lymph node. B Cas9-infected ITB monoclonal isolated colonies express an active (>95%) Cas9. In all flow cytometry experiments, we used a PD-1 guide and determined its expression before and after stimulation with IFN-γ. C Bar graph showing the number of reads per guide. Guides with the same number of reads are grouped together and indicated by the same color. Fig. S2. Knocking out TRAF3 does not affect the NF-κB pathway. A Scratch assay showing the migration of all cells over 48 h into the scratched area. Data was acquired using a live cell imaging system (Julistage). B Western blot of ITB1 and TRAF3KO lysates comparing the levels of proteins of the NF-κB pathway. The levels of p65 and RelB were monitored in the nucleus, and p100 and p52 were monitored in the whole lysates. Actin and histone H3 were used as loading controls. C Quantitative PCR shows the mRNA levels of genes related to the NF-κB pathway (RelB, IL-6, and IκBα). Cyan bars represent ITB1, solid pink bars represent TRAF3KO1, and dotted pink bars represent TRAF3KO2. D MFI of the blot on the main figure, calculated using ImageJ E Bar graph showing the differences in mean fluorescence intensity of the Cy3 channel in the nucleus between ITB1 (cyan) and TRAF3KO cells (pink). Fig. S3. B cells in TRAF3KO-injected mice express and secrete more IgAs than in ITB1-injected mice. A Expression of the costimulatory protein CD40 and interleukin-10 (IL-10) on B cells in ITB1-injected and TRAF3KO-injected mice. B, C Ig isotyping array (B) and its quantification (C). D Scatter plot showing the differentiation between the two clusters of the heatmap. E Heatmap showing the average score of each cluster for each immune pathway. F Survival curve showing the difference between patients with high B cell score [file 13046_2023_2680_MOESM1_ESM.pdf]

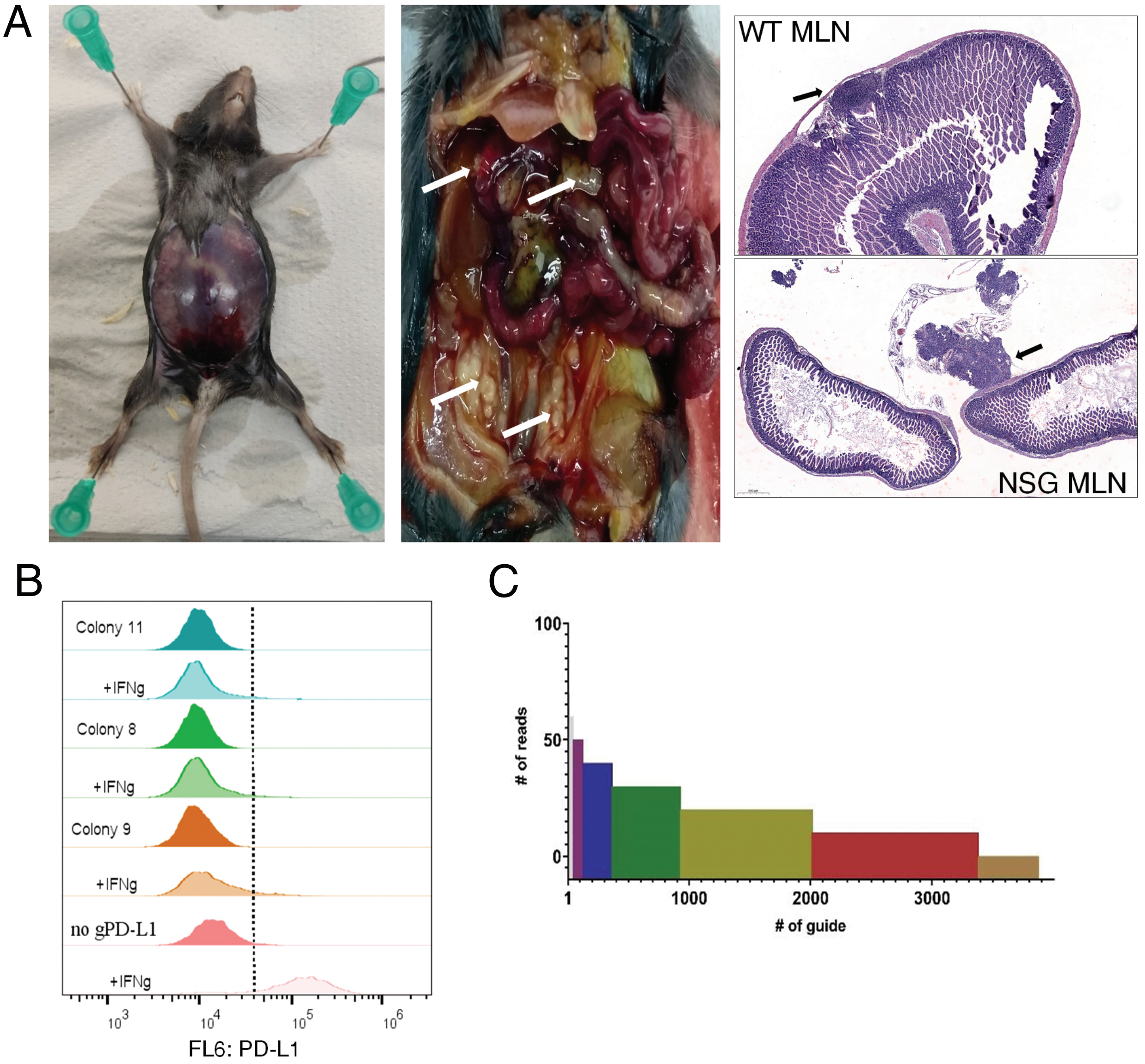
A
WT MLN
NSG MLN
B
Colony 11
+IFNg
Colony 8
+IFNg
Colony 9
+IFNg
no gPD-L1
+IFNg
10^3
10^4
10^5
10^6
FL6: PD-L1
C
100
50
0
# of reads
1
1000
2000
3000
# of guide

Figure S1

A

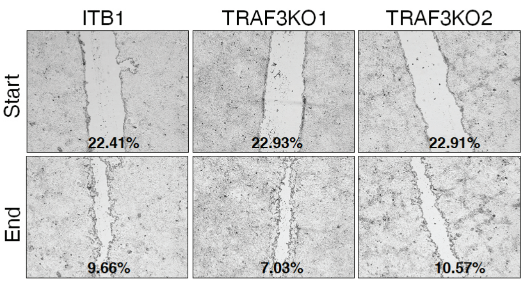

B

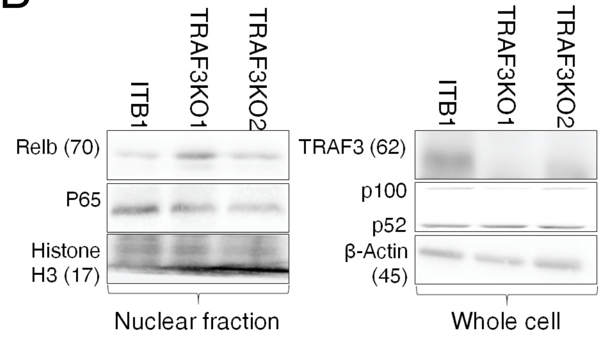

C

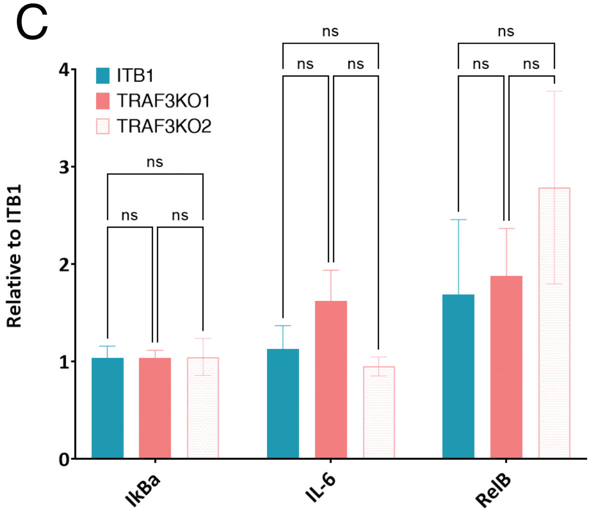

D

		ITB1	TRAF3KO1	TRAF3KO2
TRAF3	Total	313.6	0.0	0.0
	Cytoplasmic	714.7	0.0	0.0
	Nuclear	267.1	0.0	0.0
pTBK1	Total	4592.4	2011.5	2452.0
	Cytoplasmic	1225.4	2068.0	2081.5
	Nuclear	1367.8	1015.7	1097.1
TBK1	Total	1124.5	830.2	937.7
	Cytoplasmic	2993.7	4696.1	5547.5
	Nuclear	0.0	0.0	0.0
STING	Total	4334.2	6243.8	4812.9
	Cytoplasmic	910.6	1533.0	1822.5
	Nuclear	1309.7	1774.2	2377.6
β-Actin	Total	3718.6	3290.4	2661.7
	Cytoplasmic	3159.8	2467.2	2737.9
	Nuclear	2479.8	2064.1	2405.9
Histone H3	Total	816.4	608.4	562.9
	Cytoplasmic	1158.9	1107.5	438.5
	Nuclear	21651.0	22899.9	21956.8

E

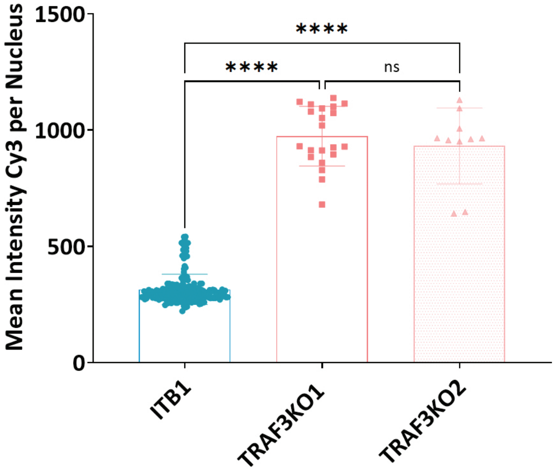

Figure S2

A

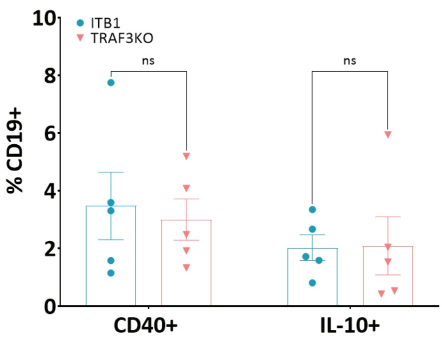

B

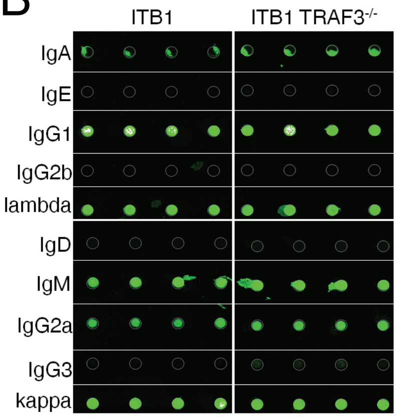

C

	P value	Mean of ITB1	Mean of ITB1 TRAF3-/-	Adjusted P Value
IgA	0.01>	2769	5157	0.01>
IgD	0.34	192.8	176.2	0.71
IgE	0.01>	60.38	87.75	0.02
IgM	0.68	16933	17703	0.71
IgG1	0.23	33466	36961	0.65
IgG2a	0.05	6398	7821	0.24
IgG2b	0.02	66.75	48.19	0.11
IgG3	0.01>	252.4	630.0	0.01>
Lambda	0.46	16645	17899	0.71
Kappa	0.02	20318	25200	0.14

D

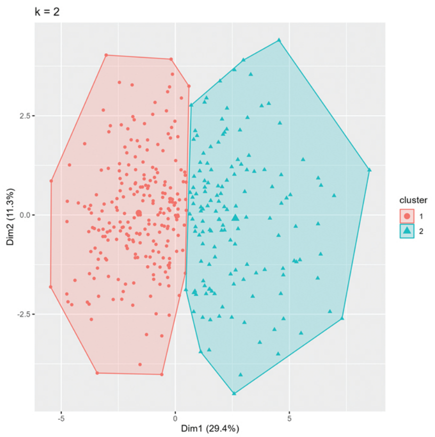

E

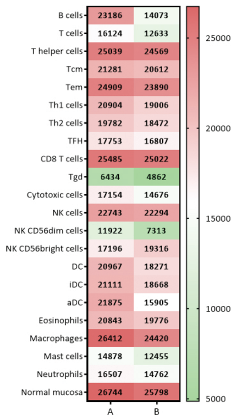

F

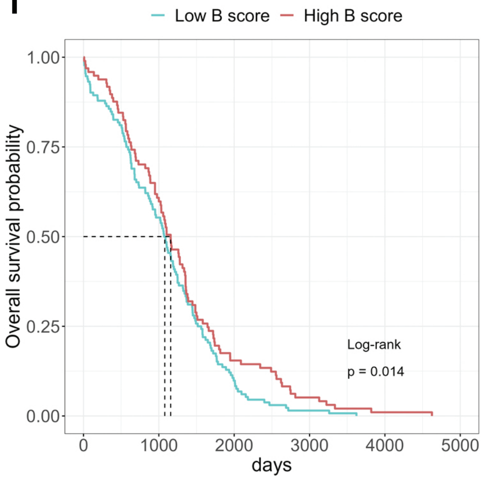

Figure S3

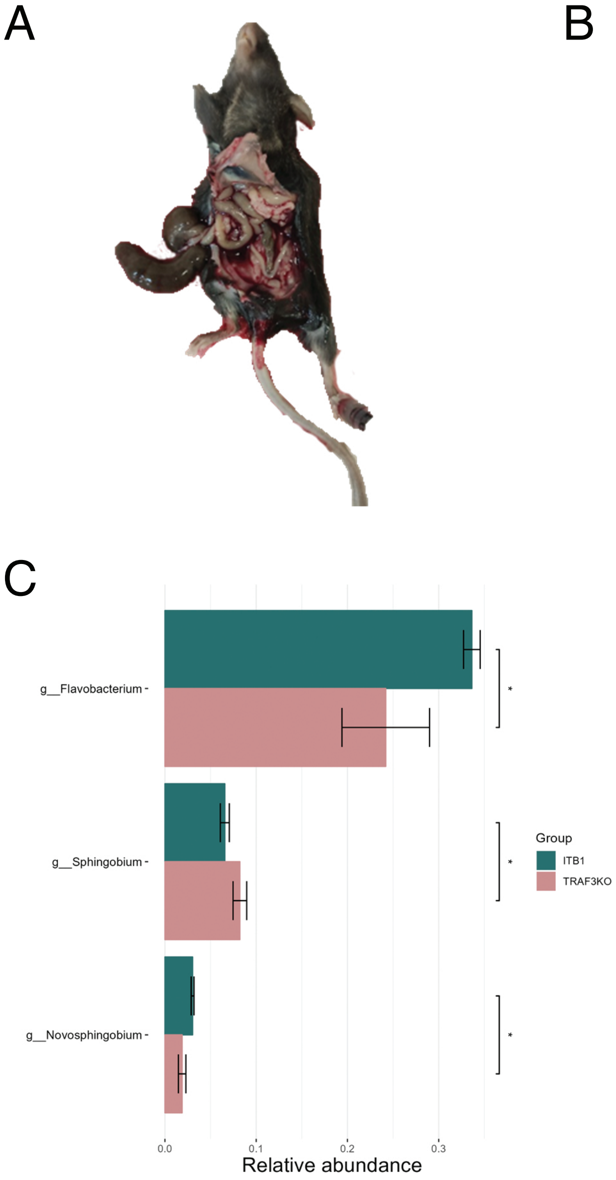
A
B
Naïve
ABX
C
g__Flavobacterium
g__Sphingobium
g__Novosphingobium
*
*
*
Group
ITB1
TRAF3KO
0.0
0.1
0.2
0.3
Relative abundance

Figure S4

A

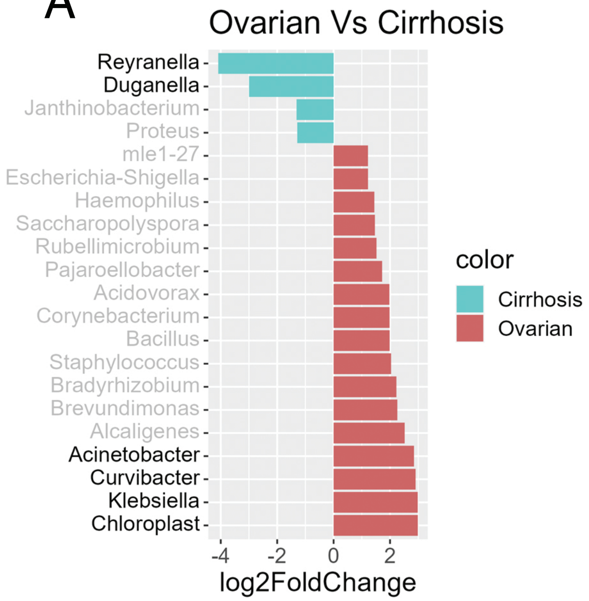

B

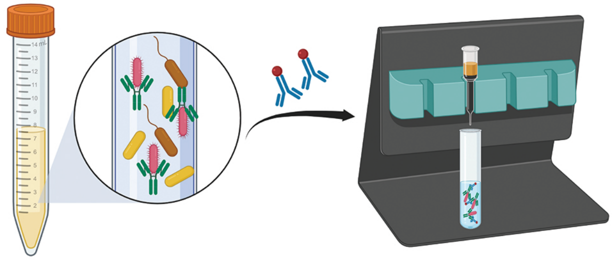

C

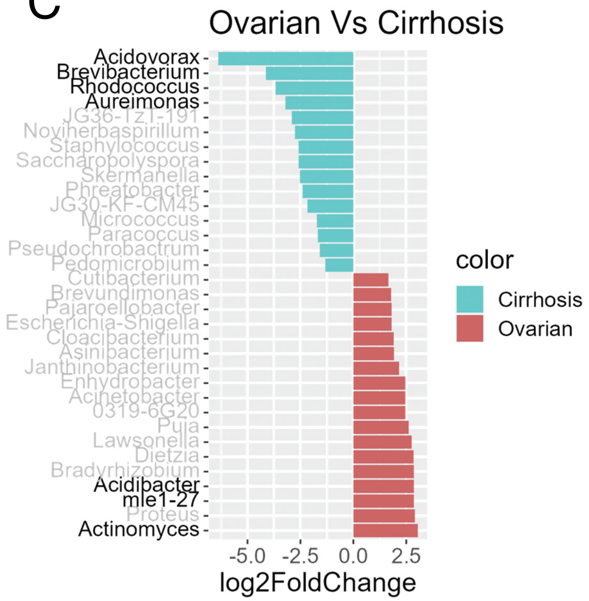

Figure S5
